# Data Quality Analyzer—Towards Optimal Radio-Frequency Frame Pair Selection for Ultrasound Elastography

**DOI:** 10.3390/bioengineering13060656

**Published:** 2026-06-03

**Authors:** Matthew Caius, Zhenbang Wang, Gregory Czarnota, Abbas Samani

**Affiliations:** 1 School of Biomedical Engineering, Western University, London, ON N6A 3K7, Canada; mcaius@uwo.ca; 2 Department of Electrical and Computer Engineering, Western University, London, ON N6A 5B9, Canada; 3 Department of Radiation Oncology Odette Cancer Centre, Sunnybrook Health Sciences Centre, University of Toronto, Toronto, ON M4N 3M5, Canada; 4 Departments of Medical Biophysics, Western University, London, ON N6A 5C1, Canada

**Keywords:** ultrasound elastography (USE), radio-frequency (RF) frames, displacement estimation, RF frame pair selection, signal decorrelation, out-of-plane motion

## Abstract

Quasi-static ultrasound elastography (USE) is a promising imaging technique for detecting malignancies by assessing tissue stiffness, but its accuracy heavily depends on the quality of radio-frequency (RF) frame pairs used for displacement estimation. A major challenge in quasi-static USE is signal decorrelation, which is primarily caused by out-of-plane motion during manual probe compression, leading to unreliable displacement fields and degraded elastography images. This paper introduces a novel, displacement estimator-agnostic method for assessing RF frame pair quality by measuring the similarity between the measured post-compression RF frame and a warped version of the pre-compression frame generated using the estimated displacement field. The proposed approach employs computationally efficient metrics such as mean squared error (MSE) and correlation, demonstrating robustness against signal decorrelation in both synthetic and clinical datasets. Additionally, we present a method to simulate realistic RF data corruption via controlled out-of-plane displacements, facilitating the development of robust motion-tracking algorithms. Validation using in silico phantoms, tissue-mimicking phantoms and clinical breast cancer and liver cancer cases confirm the method’s efficacy in identifying high-quality frame pairs, significantly improving strain image accuracy. Threshold values of 1.4 and 0.5 were determined for MSE and correlation, respectively, as being effective to differentiate between good vs. bad RF data frame pairs. This work lays the foundation for automated frame selection in USE, enhancing its diagnostic reliability and clinical utility.

## 1. Introduction

Breast, liver and prostate cancers are ones of the most common and most deadly types of cancer [1,2,3,4,5,6]. For treating these cancers, screening procedures are of the utmost importance as early detection is the key to survivability. Current screening tools include imaging modalities such as X-ray, US and MRI [7,8,9]. A major issue with most of these tools is lacking sufficient sensitivity and specificity to differentiate between benign and malignant lesions without a follow-up biopsy. One technique that can potentially address this issue is elastography, which leverages the fact that malignant lesions tend to be substantially stiffer than benign lesions [10,11,12,13,14,15,16,17]. Generally, in elastography the tissue is mechanically stimulated before resulting tissue displacement data is acquired and tissue stiffness is reconstructed. As such, elastography relies on other conventional modalities necessary to measure tissue displacement field resulting from mechanical stimulation, leading to a number of techniques such as magnetic resonance elastography (MRE). In MRE the tissue displacement field is resolved based on the MR phase data [18,19]. While MRE has high quality and high contrast, its image resolution is generally low. It is also not well suited for screening, as it requires MRI scanners in addition to special tools necessary for mechanical stimulation and data acquisition which can be cost-prohibitive in screening scenarios. Other methods to perform elastography include US techniques, namely shear elastography and quasi-static elastography and Acoustic Radiation Force Impulse (ARFI). The former uses transverse waves generated within the tissue where the wave propagation speed is measured before the tissue stiffness distribution is computed. This method requires specialized transducers working in concert with a US scanner, and it is operator independent. However, computing a stiffness image using this method relies on many assumptions that may not be accurate, hence limiting its accuracy. Quasi-static ultrasound elastography (USE) on the other hand, involves manual application of force via the US transducer. While it relies on fewer assumptions, this method introduces issues pertaining to data quality and operator dependence.

In elastography, it is well established that the quality of the stiffness image is highly dependent on the accuracy of the measured displacement field generated by motion tracking algorithms [20]. In USE, many methods have been developed for tissue motion tracking where tissue displacements are estimated by processing a pair of US RF data frames acquired at two states of the tissue pre- and post-mechanical stimulation [2,3,4,5,6,10,11]. All such methods generally assume that the imaging plane of the two RF frames is almost the same implying that there is no significant out-of-plane motion of the tissue scatterers between the frames; hence, there is little to no signal correlation. Given the manual nature of the tissue stimulation, it is hard to maintain the US probe orientation during tissue compression. As such, this assumption is rarely met and only select RF data pairs successfully fulfill this assumption.

There are three major ways of overcoming this issue. One is through improving the quality of the data by creating new techniques for acquisition where the issue of signal decorrelation is minimized. The second pursues developing displacement trackers that are robust to data with substantial signal decorrelation. Finally, the third involves assessing data quality either directly using real-time automatic data assessment tools or by allowing real-time imaging and assessing image quality. The first solution is not attractive as it involves additional data acquisition hardware such as robotic systems necessary to maintain the orientation of the US probe relative to the organ. The second method is widely pursued by integrating tissue mechanics, smoothing, multiple frame pairs and other such methods to create robust estimators [12,13,14,15,16,17,21]. In general, however, a highly robust displacement estimator is computationally intensive; hence, without extensive optimization, such estimators are difficult to use in clinical settings. The final solution of real-time assessment of data allows clinicians to assess data in a far more convenient way without deviating from the conventional procedure of US imaging [22]. In this paper we propose a new method by which automatic quality assessment of RF data pairs can be performed efficiently, allowing for a quantitative measure of data quality.

There are currently very few methods proposed to assess the quality of pairs of RF data frames acquired for tissue motion tracking. These methods are typically not agnostic to displacement estimators; hence they cannot be used independently from the underlying methodology of the estimator. For instance, PCA-GLUE was recently proposed for data quality assessment [23]. While it stands on solid foundations of machine learning, this quality assessment model was developed using data pertaining to tissue mimicking phantoms and only three patients; thus, it may not be rigorous for clinical applications. A major objective of this work is to develop a displacement estimator agnostic methodology for assessing the quality of the displacement field obtained in USE in terms of signal decorrelation. Ideally, the method should provide near real-time performance to be useful as a data analyzer tool at the bedside. A less desirable but useful alternative involves automatic agnostic assessment of displacement quality, which enables rapid analysis of a US RF-video after data acquisition. This alternative relaxes the requirement for real-time operation, making the use of more computationally intensive displacement estimators more feasible. Moreover, it circumvents the requirement of tool installation on the US scanner if the RF data can be exported from the machine. These features make the clinical convenience and feasibility of the technique more appealing. To provide a proper control protocol for this investigation, this paper is also geared towards the objective of introducing a way of purposefully contaminating RF data to simulate the effects of signal decorrelation. This data contamination method can also be used in the development of displacement tracking algorithms by checking their robustness against known data corruption. To achieve this, a dataset pertaining to tissue-mimicking phantoms was created in silico using finite element modelling (FEM) and an ultrasound computer simulator (FIELD II ultrasound simulation software [24,25]). This data was generated to simulate various levels of data corruption generated by out-of-plane movement of scatterers. This data contamination method can be employed for validating displacement estimators using data of more realistic quality, rather than relying on clean synthetic data samples [12,14,26,27,28,29]. This will allow for the development of more robust displacement estimators through quantifying the robustness of developed algorithms against out-of-plane displacement.

In short, this paper seeks to provide two major contributions: (1) developing a displacement estimator-agnostic methodology for assessing the quality of tissue displacement and (2) providing a method for contaminating in silico RF data that mimic signal decorrelation resulting from out-of-plane displacements to generate more realistic data. The latter serves as a controlled simulator of signal decorrelation necessary for developing robust motion tracking methods. The methods proposed in this paper form the basis of developing techniques for optimal selection of RF data frame pairs aimed at generating high-quality USE images.

## 2. Materials and Methods

### 2.1. Overview of Proposed Method

The proposed method of displacement quality assessment assumes that RF signal decorrelation is the main contributor to the degradation of estimated displacement field. The main source of this decorrelation is out-of-plane displacement and nonuniform compression of the tissue during tissue mechanical stimulation in US elastography. The proposed assessment method is based on the premise that an accurate displacement field estimated using its two corresponding pre- and post-deformation RF frames can be used to warp the pre-deformation RF frame to estimate the post-deformation RF frame with high accuracy. In other words, using an inaccurate displacement field to warp the pre-deformation RF frame leads to a mismatched post-deformation RF frame. As such, to assess the accuracy of the displacement field, the ability of this field to regenerate the measured post-compression RF data frame can be assessed. This can be performed by measuring the similarity of the pre-deformation RF frame warped using the displacement field with the measured post-compression RF frame used to estimate the displacement field. The higher this similarity the better the displacement field estimate.

### 2.2. Evaluation Algorithm

Assuming that $S_{1}$ and $S_{2}$ represent the US scatterers’ distribution at two states of pre- and post-deformation, and $d_{x},\;d_{y}\;and\;d_{z}$ describe the true displacement components of the scatterer $S_{1}$ at (*x*,*y*,*z*) post-deformation, the following equation can be written.

(1) $$\begin{array}{c} S_{2}\left(x+d_{x},y+d_{y},z+d_{z}\right)=S_{1}\left(x,y,z\right) \end{array}$$

This equation leads to the following if no decorrelation resulting from out-of-plane displacements of the scatterers ($d_{z}$ = 0) is assumed:

(2) $$S_{2}\left(x+d_{x},y+d_{y}\right)=S_{1}\left(x,y\right)$$

The RF frame is a function F of $S_{1}$ and $S_{2}$ distribution, where F describes the scatterers’ interaction under ultrasound stimulation, and *I*_1_ and *I*_2_ denote the pre- and post-deformation RF data frames, *I*_1_ = *F*(S_1_) and *I*_2_ = *F*(S_2_). Assuming small scatterer displacements, the following can be written:

(3) $$I_{2}\left(i+d_{i},j+d_{j}\right)≅I_{1}\left(i,j\right)$$

In the above, $d_{i}$ and $d_{j}$ denote the displacement components of a scatterer located at the center pixel (i,j) location corresponding to (*x*,*y*) in *I*_1_. The proposed method is motivated by the hypothesis that the closer the estimated displacement field in satisfying Equation (3), the more accurate. This is a well-motivated hypothesis as many displacement estimators already consider a version of Equation (3) when generating the initial displacement estimate [14,21,26,30,31,32]. To evaluate this metric, the forward displacement operation of Equation (3) is performed on $I_{1}$ to compute the simulated $I_{2}$ ($I_{2warp}$) before comparing it to the measured $I_{2}$ ($I_{2meas}$) using proper similarity measures. This forward operation is achieved by considering the estimated displacement field as a geometric operation and performing image warping to generate the signal produced by the combination of $I_{1}$ and the displacement field to generate $I_{2warp}$, which should be identical to $I_{2meas}$ in an ideal scenario. This method uses computationally inexpensive metrics and processes through posing the problem as a geometric operation.

### 2.3. Algorithm Performance Assessment with in Silico Phantom

**Figure 1:**
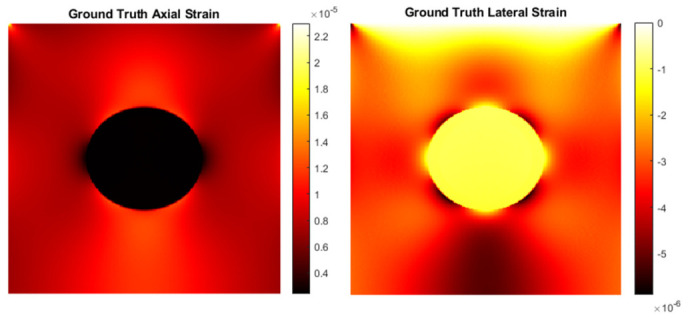
Typical “ground truth” axial and lateral strain images of a computational block-shaped phantom generated by the FE solver with Young’s moduli X and Y (X > Y) for the inclusion and background, respectively.

To develop the algorithm, we initially conducted a study using simulated data. For this purpose, we utilized the FIELD II ultrasound simulator along with an FE solver developed in our laboratory to generate the “ground truth” displacements of the computational block-shaped phantom shown in Figure 1, which results from simulation of a US probe compressing applied to the top surface of the phantom. The FE solver was developed in C++ before it was rigorously tested for accuracy. The inclusion and background tissue parts have Young’s moduli denoted by X and Y, respectively, with X > Y. The RF data was computationally generated by creating a random field of scatterers and solving the wave equation for the field of scatterers using Field II to generate the first RF frame $I_{1}$. Then, using the “ground truth” displacement field obtained through the FE solver, the scatterers were moved according to the displacement field at their location to generate the second RF frame $I_{2}$.

To corrupt the data of the first RF frame $I_{1}$ with out-of-plane movement, a variable amount of out-of-plane displacement was added from a Gaussian distribution with 100 standard deviations ranging from 0 to 15 mm, where 0 represents perfect data with no out-of-plane displacements. The forward model described earlier was used to warp frame $I_{1}$ to generate the second RF frame $I_{2wrap}$. The displacement field corresponding to each generated RF data pair, which was obtained based on a set level of out-of-plane displacement, was computed using the analytic minimization in 2D (AM2D) technique [31]. While this method was used, the RF data pair quality assessment presented here is independent of the displacement estimator. As potential measures for accuracy and similarity of $I_{2wrap}\;$with the target $I_{2meas}$, the MSE, Correlation, CNR and SNR for each amount of corruption were recorded and analyzed. In this case, the metrics will be henceforth referred to as “warped” metrics. For qualitative assessment of RF data pairs, corresponding strain images generated from the displacement estimation were visualized for inspection of the degradation of quality.

One may speculate that assessing the correlation between unwarped *I*_1_ and $I_{2meas}$ may be sufficient for assessing the quality of RF data frame pair while being computationally less expensive. To test this hypothesis, we also calculated the correlation and MSE metrics of $I_{2wrap}$ and the unwarped I_1_, which was corrupted by the same variable levels of out-of-plane displacements. The metrics obtained through this procedure without warping will be henceforth referred to as “naïve” metrics.

### 2.4. Algorithm Performance Assessment on Tissue-Mimicking Phantom RF Dataset

To further assess the robustness of the proposed method under realistic clinical conditions, the proposed algorithm was evaluated using a tissue-mimicking phantom RF dataset [14,33,34]. This dataset comprises 2294 RF frame pairs acquired from various regions of a CIRS phantom using an Alpinion E-Cube R12 research ultrasound system (Bothell, WA, USA). Unlike continuous RF video, this dataset was provided as pre-paired pre- and post-compression frame pairs. This dataset was experimentally acquired, employs a different phantom model and a different ultrasound machine, and consists of real freehand scans that naturally contain decorrelation and out-of-plane motion. Accordingly, the correlation and MSE were computed for all 2294 frame pairs, and their distributions were analyzed to verify the transferability of the thresholds adopted in this paper to large-scale real data. The top three and bottom three frame pairs were also compared.

### 2.5. Algorithm Performance Assessment with Clinical Data

To test the viability of this method in the clinic, RF videos of clinical breast cancer cases were analyzed pair-by-pair using the proposed method to find the best and worst frame pairs. The pertinent patient study was conducted in accordance with institutional research ethics board approval from Sunnybrook Health Sciences Centre, Toronto, Canada. The top five and bottom five pairs, which were ranked based on their respective MSE and correlation metrics, were visualized and manually assessed and compared to validate the capabilities of the proposed algorithm. Histograms and tables for MSE and Correlation were also generated for these clinical cases. The clinical effectiveness of the proposed algorithm was further validated using RF data acquired for four liver cancer patients from another dataset acquired in accordance with institutional research ethics board approval from Johns Hopkins University, Baltimore, MD, USA [30]. The proposed algorithm was run for each patient, and the resulting similarity metrics and strain maps were computed.

## 3. Results

### 3.1. “Warped” Metrics of RF Data Frame Pairs in In Silico Phantom

**Figure 2:**
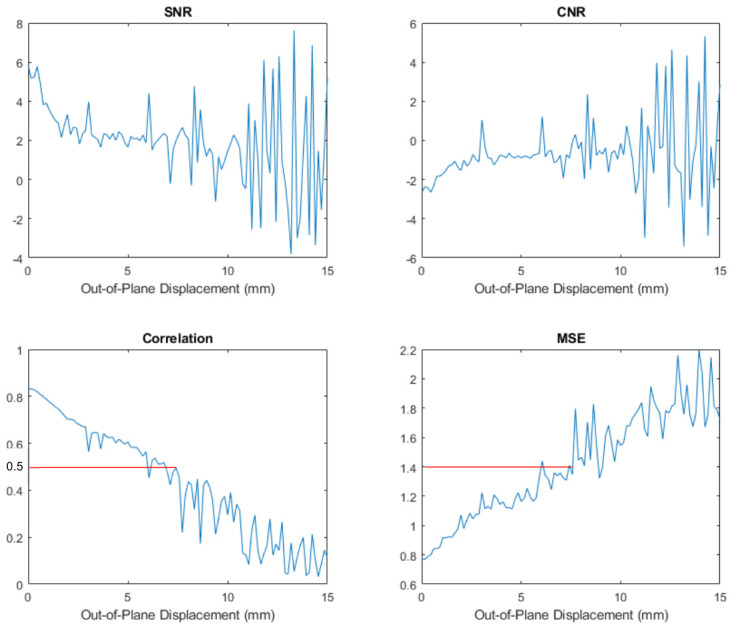
Metrics of RF data pair quality as the out-of-plane displacement increases (standard deviation of out-of-plane displacement in mm) for the synthetic data. As the out-of-plane corruption increases, SNR and CNR become progressively unreliable, while MSE and Correlation scores become progressively inferior. The threshold values of 0.5 and 1.4 of the latter two metrics where the oscillation is intensified are shown.

Figure 2 illustrates the CNR, SNR, Correlation and MSE metrics for each corrupted synthetic data sample generated as described earlier, which henceforth is referred to as “warped” metrics. As expected, this figure shows that as corruption increases, the metrics proposed for quality assessment decrease. As can be seen, the correlation and MSE worsen with corruption, and CNR and SNR become noisier with higher levels of data corruption. Among the four metrics, CNR and SNR exhibit highly oscillatory behaviour at high levels of decorrelation with no clear increasing or decreasing trend, indicating low reliability for RF frame pair quality assessment or selection. Interestingly, at Correlation and MSE values of 0.5 and 1.4, respectively, and beyond, high amplitude oscillation in the respective metrics is observed, leading to the selection of these values as threshold for differentiation between good and bad RF data pairs.

**Figure 3:**
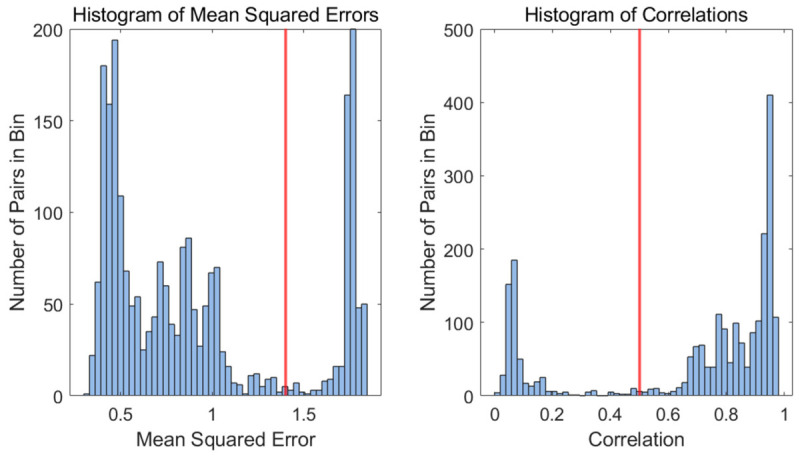
Histograms of MSE (**left**) and Correlation (**right**) generated for the tissue-mimicking phantoms. These histograms show bimodal distribution where the threshold values of 0.5 for Corr and 1.4 for MSE that (shown by red lines) fall precisely at the center of their respective valleys, lending further credibility to their validity in distinguishing between good vs. bad RF frame pairs.

To further assess the accuracy of the Correlation and MSE threshold values of 0.5 and 1.4 beyond simulated data, we plotted the distribution histograms of warped Correlation and warped MSE for the tissue mimicking RF data as shown in Figure 3. The left panel in the figure shows the histogram of warped Correlation where two clearly separated peaks are visible. The red solid line marks the threshold of 0.5 adopted based on the initiation of oscillatory behaviour. The right panel shows the histogram of warped MSE. The red solid line denotes the threshold of 1.4 adopted based on the initiation of oscillatory behaviour. The key observation is that both threshold values determined based on the simulated data fall precisely at the center of their respective valleys. The pass rate corresponding to the warped correlation and warped MSE are 1719/2294 = 74.9% and 1740/2294 = 75.9%, respectively. This analysis lends further credibility to the threshold values of 1.4 and 0.5 previously obtained for the warped MSE and warped correlation, respectively.

**Figure 4:**
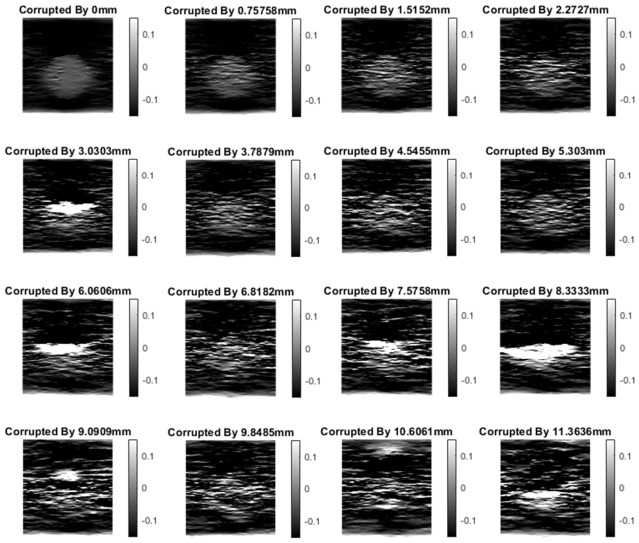
Progressive degradation of axial strain image quality and increased artefacts with increasing out-of-plane displacement data corruption of synthetic phantoms is shown. The artefacts appear in the form of unrealistically high-strain (both compressive and tensile) areas spreading out progressively with increased out-of-plane displacement.

Axial strain images were computed for various levels of out-of-plane displacement corruption. Figure 4 illustrates the progressive degradation of the generated axial strain images derived from the corrupted RF data. This degradation was investigated theoretically through Fourier analysis and experimentally through analyzing the results of the tissue mimicking phantom dataset. Both consistently showed that the estimated strain grows substantially with the level of out-of-plane corruption.

### 3.2. “Naïve” Metrics of RF Data Frame Pairs in In Silico Phantom

**Figure 5:**
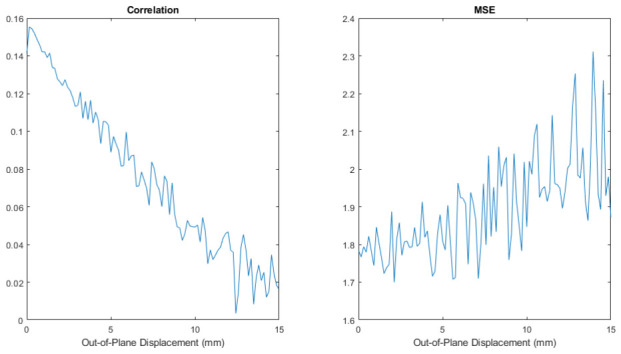
Naïve correlation and MSE metrics show a similar pattern to their warped counterparts where correlation and MSE become progressively inferior with increased out-of-plane displacement. Compared to its warped counterpart, the the naïve MSE exhibits onset of high oscillation with lower level of data corruption. With the naïve correlation, the values are less convincing, as the correlation starts at 0.16. Therefore, warped correlation is a much more robust method than naïve correlation.

Figure 5 illustrates the variations of the naïve correlation and MSE metrics vs. out-of-plane displacement. As can be seen in this figure, these parameters show a similar pattern; however, the magnitudes of these parameters are much less desirable as they cover a small dynamic range of values compared to the warping-based method, while the decrease is far noisier. As such, it can be concluded that the warping-based method is superior.

### 3.3. RF Data Pair Quality Assessment of Tissue-Mimicking Phantom Dataset

**Figure 6:**
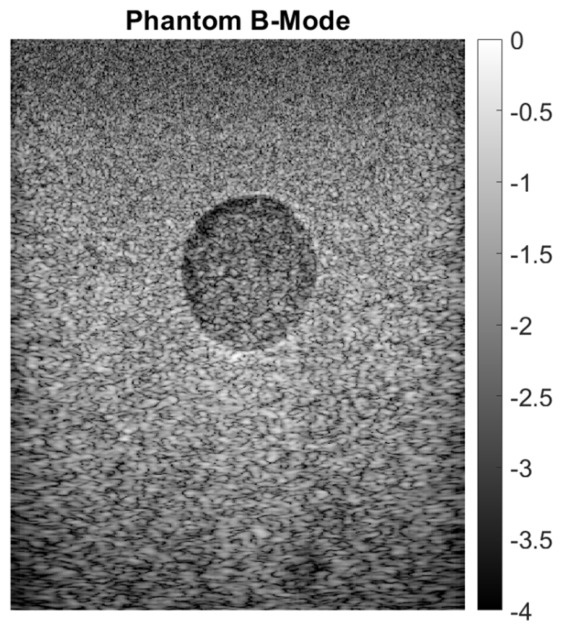
B-Mode image of the CIRS phantom with the hard inclusion.

**Figure 7:**
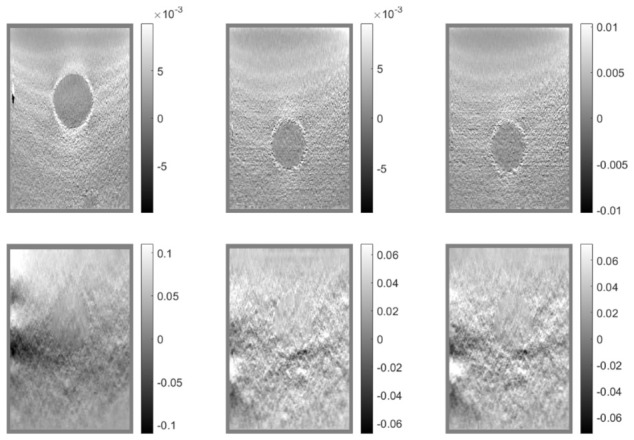
Phantom example where strain images corresponding to the top three ranked frame pairs (**top**) and bottom three ranked frame pairs (**bottom**) are shown. As can be seen, the bottom three frame pairs are inferior compared to the top three, where the hard inclusion is clearly delineated in the top three strain images but indistinguishable in the bottom three.

Figure 6 illustrates a B-mode image of a sample tissue-mimicking phantom in the dataset, in which a hard inclusion is clearly visible at the center of the image. Figure 7 compares the top three and bottom three ranked frame pairs of this dataset. In the high-scoring strain images, the boundary of the hard inclusion is clearly visible, whereas the bottom three strain images are noisy and the inclusion cannot be distinguished.

**Figure 8:**
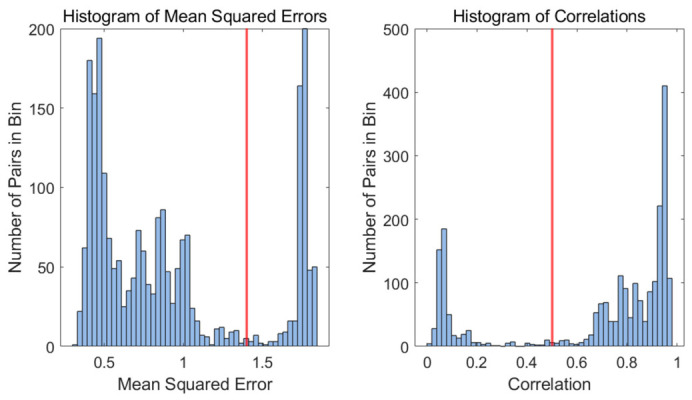
Histograms of the mean squared error and correlation coefficients for all 2294 RF data pairs of the phantom dataset. The red lines indicate the threshold values of 1.4 and 0.5.

Figure 8 presents the distribution of the similarity metrics as histograms. Both metrics exhibit a distinctly bimodal distribution. Specifically, for the correlation distribution, the low-quality cluster has a mean value of approximately 0.08 while the high-quality cluster has a mean value of approximately 0.95, with a valley in between.

For the warped MSE distribution, the high-quality cluster has a mean value of approximately 0.7 while the low-quality cluster has a mean value of approximately 1.8, with the valley located between approximately 1.3 and 1.6. The thresholds adopted in the paper based on synthetic data fall almost exactly at the center of the natural valley observed in this real hand-scanned data. This test validates the appropriateness of the paper’s thresholds on experimental data entirely independently from the synthetic data used in this work. Applying the determiend threshold values, 74.9% (1719/2294) of the 2294 RF frame pairs have a Correlation ≥ 0.5 and 75.9% (1740/2294) have a warped MSE ≤ 1.4, yielding very similar acceptance rates for the two metrics. This indicates that, on large-scale experimental data, the determined threshold values consistently classify approximately three-quarters of the frame pairs as usable.

### 3.4. RF Data Pair Quality Assessment of Clinical Data

**Figure 9:**
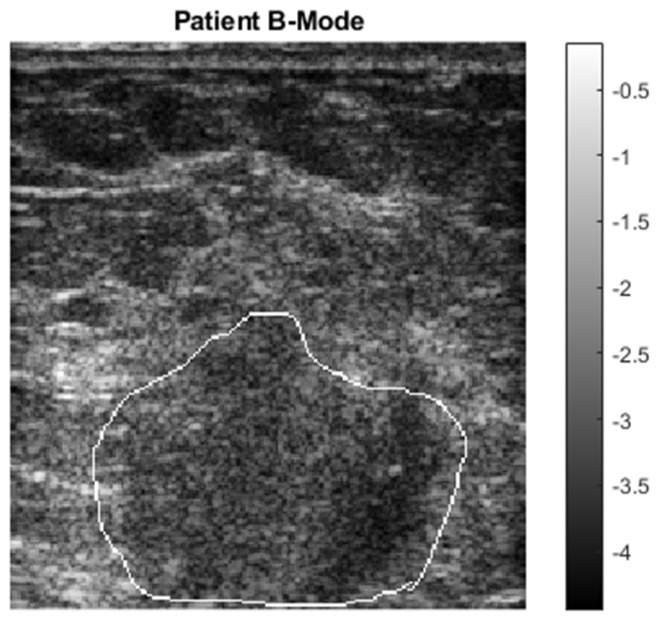
B-Mode image of breast cancer Patient 1 where the tumour is outlined by a clinician.

**Figure 10:**
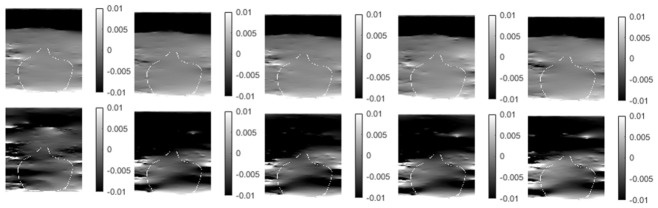
Clinical example of Patient 1 where strain images corresponding to top five ranked frame pairs (**top**) and bottom five frame pairs (**bottom**) are shown. As can be seen, the low bottom five frame pairs are inferior compared to the top five. The tumour boundary is outlined in white based on the segmentation of the B-mode image.

**Figure 11:**
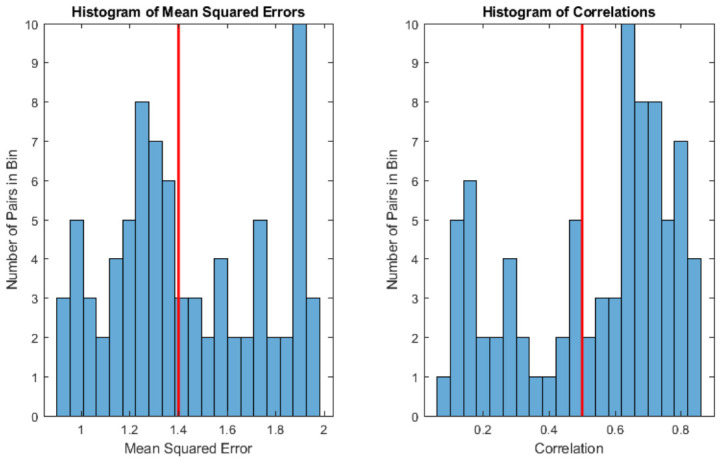
The histogram of mean squared error and correlation coefficients for each RF data pair of Patient 1. For this example, most pairs are at least passable in terms of correlation coefficients, with the acceptance threshold taken at the determined MSE and Correlation values of 1.4 and 0.5 that are shown by the red lines, respectively.

**Figure 12:**
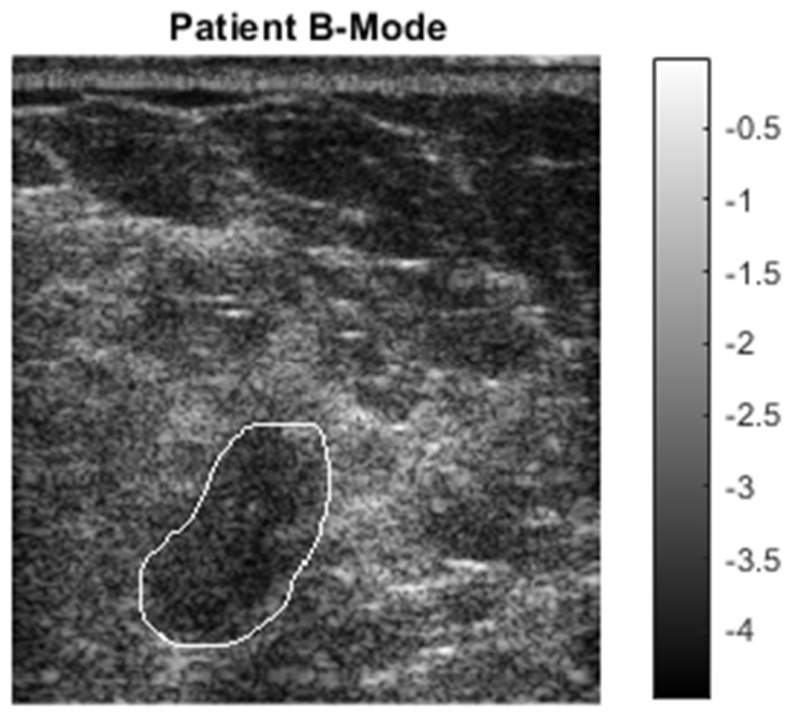
B-Mode image of breast cancer Patient 2 where the tumour is outlined by a clinician.

**Figure 13:**
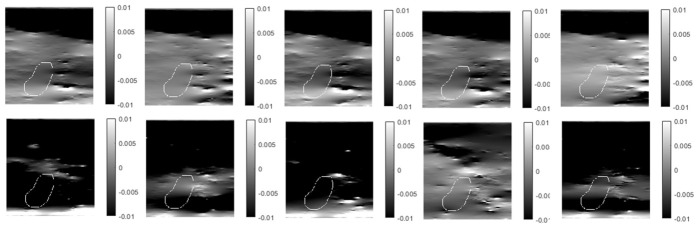
Clinical example of Patient 2 where axial strain images corresponding to top five ranked frame pairs (**top**) and bottom five frame pairs (**bottom**) are shown. As can be seen, the low bottom five frame pairs are inferior compared to the top five. The tumour boundary is outlined in white based on the segmentation of the B-mode image.

**Figure 14:**
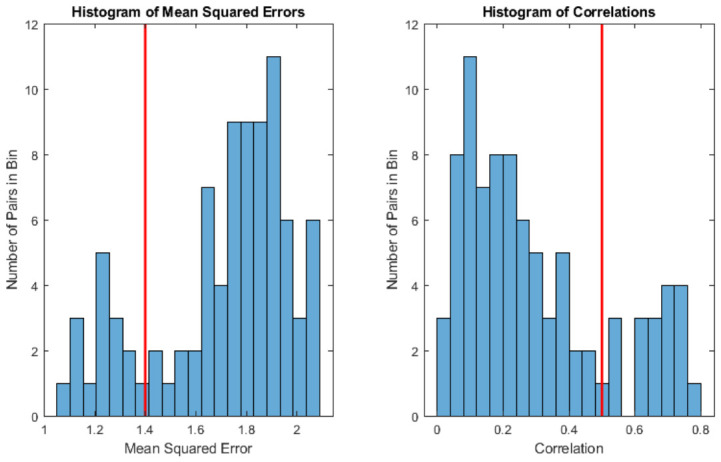
The histogram of mean squared error and correlation coefficients for each pair in clinical example 1. For this particular example, with the acceptance threshold values of 0.5 and 1.4 that are shown by the red lines, most pairs are of very low quality and should not be used for elastography.

Figure 9 illustrates the B-Mode images of the first breast cancer patient with the tumour outline obtained after image segmentation by a radiologist. For this clinical case, Figure 10 compares the top five ranked frame pairs with the bottom five frame pairs. In these figures, the tumour outlines are overlaid on the axial strain images. As can be seen, the top five frame pairs are far smoother and provide a much less obstructed view of the strain. In Figure 11 the histograms for all valid frame combinations can be seen. As seen in this figure, the RF data pair acceptance threshold was taken at the determined MSE and Correlation values of 0.5 and 1.4, respectively. Figure 12 illustrates the B-Mode image of the other breast cancer patient with the tumour outline obtained after image segmentation by a radiologist. For this patient, Figure 13 compares the top five ranked frame pairs with the bottom five frame pairs where, similar to the first patient, it can be seen that the top five frame pairs are far smoother and provide a much less obstructed view of the strain. Figure 14 illustrates the histograms for all valid frame combinations for the second breast cancer patient where the same RF data pair acceptance threshold values of 0.5 and 1.4 are shown. Figure 11 shows that a larger proportion of frame pairs correspond to high correlation whereas Figure 14 indicates a large bias towards low-quality frame combinations.

Overall, as shown in Table 1, the top five frame pairs have overwhelmingly superior Correlation and MSE measures compared to the bottom five frame pairs, showing a high degree of contrast in the measures, especially with correlation coefficients, which range from 0 up to 0.8+ (Figure 10 and Figure 13). These results lend further credibility to the proposed technique in the clinic.

**Figure 15:**
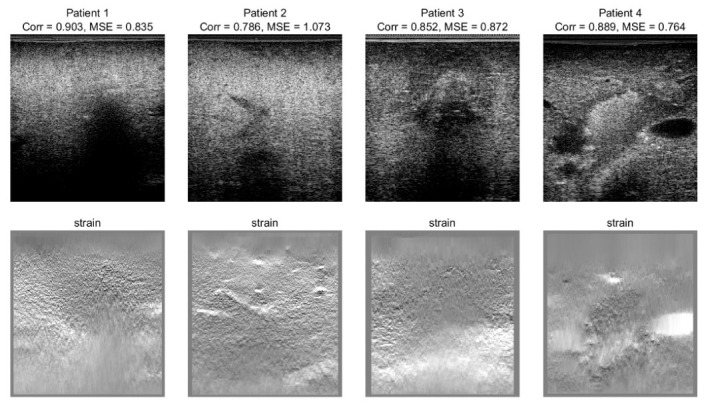
Clinical examples of four liver cancer patients [30]. **Top** row: B-mode images with the corresponding correlation and MSE values. **Bottom** row: AM2D strain maps.

For each of the four liver cancer patients where only a predetermined RF data pair is available, the resulting similarity metrics and strain maps are shown in Figure 15. For these patients, the correlation values ranged from 0.786 to 0.903 and the warped MSE values from 0.764 to 1.073, all satisfying the thresholds. Visually, the AM2D strain maps of all four patients exhibit clear texture. The proposed algorithm produced stable and reasonable quality scores on all four cases, further supporting the effectiveness of the method in real clinical scanning scenarios.

All experiments were performed on CPU (AMD Ryzen 7, Windows 11, MATLAB R2024a). Runtime was measured at four resolutions, ranging from a small 1000 × 64 to a large 5000 × 256, with 2500 × 256 corresponding to the resolution used in the manuscript. The performance target was the 30 fps real-time budget, i.e., no more than 33.33 ms per frame pair.

We benchmarked the algorithm on a single-threaded CPU implementation at four resolutions (1000 × 64 to 5000 × 256), with results summarized in Table 2. The results indicate that the algorithm exhibits perfect linear scaling. At the resolution used in this article (2500 × 256), the per-pair end-to-end latency was 49.8 ± 1.7 ms, corresponding to 20 fps, well within the typical 15–30 fps operating range of commercial clinical ultrasound scanners. When the resolution was reduced in the lateral direction to 2500 × 128, the latency dropped to 26.5 ms per pair (38 fps), comfortably exceeding the 30-fps real-time budget.

For further assessment of the proposed technique, we examined how other similarity metrics vary as out-of-plane displacement (σ) is swept from 0 to 15 mm; the results are summarized in Table 3. The table shows that warped Correlation exhibits a span of 0.70, occupying 70% of the [0, 1] range; warped MSE and the combined metric similarly exhibit span values of 3.5 and 4.0, respectively. SSIM, by contrast, remains confined to 0.90 and 0.99, spanning only 0.09, which is less than 9% of its range, i.e., roughly one-seventh that of Corr. This gap has direct implications for clinical robustness: with the Correlation threshold of 0.5, the worst frame (0.30) and the best frame (1.00) are clearly separated; with SSIM, a typical good clinical frame sits at ~0.98 and a bad frame at ~0.93, leaving a decision margin of only 0.05.

The measured per-call latency of each metric at the resolution 2500 × 256 is summarized in Table 4, which indicates that warped Corr and warped MSE require approximately 2.6 ms and 1.6 ms, respectively (a combined cost of less than 4.2 ms), NCC about 4 ms, MI 11.7 ms and SSIM 24.7 ms, indicating that SSIM is roughly 10× more expensive than Corr per call.

## 4. Discussion

The proposed technique aims at quality control of an estimated displacement field of tissue in USE. It examines the suitability of the RF data frame pair of $I_{1meas}$ and $I_{2meas}$ used to estimate the displacement field through assessing the pair signal decorrelation. This is performed by measuring the similarity between $I_{2meas}$ with $I_{2wrap}$ computed by combining $I_{1meas}$ with the estimated displacement field according to Equation (3). The linear elasticity assumption used in calculating the displacement field of the in silico phantoms is justified by the small deformations typically induced during tissue stimulation in elastography. Moreover, although assuming a uniform scatterer density is common in ultrasound simulation, incorporating nonuniform scatterer distributions is anticipated to improve the simulation accuracy. Another fundamental assumption used in this study is that speckle decorrelation is entirely attributed to our-of-plane displacement while perfusion, which is related to microvascular flow, is ignored. Between the two, the former is known to be the dominant contributor (often by one order of magnitude) to speckle decorrelation. Unlike other relevant methods developed using machine learning for RF data pair quality assessment which are displacement estimator-specific [23], the proposed method requires no manual data labelling and is displacement estimator-agnostic. As such, a potential application of the proposed method is its utility during the development stage of new tissue motion tracking techniques as the method can be used with any displacement estimation methodology.

Results obtained in this investigation have shown that the technique is quite sensitive to out-of-plane displacement, which is a major contributor to signal decorrelation common during data acquisition in USE. They indicate that both the correlation and MSE metrics, which are used to measure the similarity between $I_{2wrap}$ and $I_{2meas}$, exhibit clear variation with increasing out-of-plane corruption in the synthetic data sample, while the CNR and SNR metrics proved unreliable in quality assessment of RF data frame pairs. It is interesting to note that when the out-of-plane displacement increases, it is not simply a degradation of the SNR, but in fact as seen in Figure 4, there are increasingly larger and more unrealistically high-strain regions in pertinent strain images. The reason for this is the amplified oscillatory behaviour of the strain since it is calculated as the spatial derivative of the displacement field, hence the displacement tracking algorithms are led astray, leading to regions of unrealistically high strain both compressive and tensile. This is confirmed through Fourier analysis and experimentally through analyzing the results of the tissue-mimicking phantom dataset. Such high strain distribution is physically impossible under quasi-static loading. This explains why CNR and SNR values do not simply decay but lead to extremely high or extremely low values. More investigation into this may yield new ways to address the issue of out-of-plane strain. In addition to quality control of displacement field, the proposed method may allow for automatic or semi-automatic determination of calibration parameters used in tissue motion tracking algorithms through optimizing the correlation and MSE metrics. This can be more effective than current methods that determine these parameters using ad hoc methods [13,14,26,30,31]. The standard similarity measure values used in this investigation were determined at 0.5 and 1.4 for Correlation and MSE, respectively. This was based on initiation of large oscillation in the two similarity metrics beyond the determined values of 0.5 and 1.4 as evident in Figure 2. The reliability of these values was evaluated based on an experimental hand-scanned tissue-mimicking phantom dataset. It was observed that both the correlation coefficients and the MSE followed a clearly bimodal distribution, which separated the frame pairs into a high-quality cluster and a low-quality cluster. The values of 0.5 and 1.4 fell almost exactly within the natural valley between these two clusters. This indicates that the threshold values are not simply arbitrary values tied to the synthetic data, but instead correspond to a genuine boundary between usable and unusable frame pairs. The two metrics also accepted nearly the same proportion of pairs, which shows that the chosen values do not introduce a noticeable bias between the two measures.

Aiming at improving the realism of data generated through in silico methods, another objective of this investigation is to introduce a new method to simulate data decorrelation due to tissue mechanical stimulation issues to generate in silico synthetic data. As illustrated in Figure 4, the proposed method can realistically simulate RF data decorrelation resulting from out-of-plane displacement, which is confirmed by the progressive decrease in image quality as out-of-plane displacement increases. The same trend is observable in clinical data as shown in Figure 10 and Figure 13, where similar artefacts, which include areas of unrealistic high strain regions in the bottom row, are seen. Seeing similar artefacts in both the simulated and clinical examples is a good indication that the corruption method can be used to inform algorithmic design, as it appears to be capable of simulating some artefacts.

As stated earlier, a bottleneck in using the proposed method of RF data frame pair selection is the lack of data pertaining to proper similarity measures threshold. In this investigation, values of 0.5 and 1.4 were determined carefully for Correlation and MSE, respectively. For effective clinical utility, this needs to be determined based on a comprehensive dataset. With the absence of more reliable threshold values, the technique can be at least employed to narrow down the search window significantly by discarding obviously bad frame pairs with unfavourable similarity measure values. Moreover, results obtained in this investigation may point to criterion which may be employed as a second line of defense against false positive or negative diagnosis outcomes. For example, Figure 4, Figure 10 and Figure 13 include artefact regions in the shown strain images that point to very soft or unrealistically heterogeneous tissue. The presence of inclusion with such characteristics in a strain image combined with corresponding low similarity measures may confirm selecting a wrong RF data frame pair. To automate this assessment process, filtering techniques can be developed to disregard frames which include non-physiologically high-strain values. Similarly, measures of noise, such as Laplacian norm of the displacement field, or the divergence of the displacement field may be utilized for eliminating improper RF data frame pairs. Another avenue could be to design a deep-learning-based displacement estimator that simultaneously estimates the displacement field and labels it as either suitable or unsuitable [5,6,12,27,33,35,36].

Other than laying a foundation for automatic RF data frame pair selection in USE, the proposed technique can be applied to provide clinicians with a real-time objective measurement of the quality of the observed strain image, which can be highly valuable in determining if an inclusion in the image is an artefact or indeed a tumour. By leveraging metrics that are already used in the calculation of the displacement estimate, this method can be made computationally free by simply saving and outputting intermediate values from the tissue motion tracking algorithm, which means that it would not impact the usability of any imaging system it is incorporated in. This development aims at improving the accuracy of USE through systematic selection of RF data frame pair which is an essential prerequisite for USE image quality assurance. It may contribute to further establishment of USE for clinical use.

## Figures and Tables

**Table 1:** Mean MSE and Correlation for top five and bottom five frame pairs for Patients 1 and 2. As can be seen, there is a considerable difference in the quality metrics between the two groups.

Clinical Example	Mean MSE Top Five	Mean MSE Bottom Five	Mean Correlation Top Five	Mean Correlation Bottom Five
1	0.95	1.93	0.83	0.12
2	1.12	1.95	0.75	0.04

**Table 2:** Per-pair runtime and throughput at four resolutions (CPU, mean over fifty timed runs after five warmups).

	Mean (ms)	Std (ms)	Median (ms)	Throughput (Hz)
1000 × 64	4.09	0.35	3.98	245
2500 × 128	26.45	0.90	26.28	38
2500 × 256	49.83	1.67	49.40	20
5000 × 256	110.56	2.55	109.98	9

**Table 3:** Dynamic range of similarity metrics on synthetic data (σ from 0 to 15 mm).

Metric	Good Frame (σ = 0)	Bad Frame (σ = 15 mm)	Span
warped Corr	1.00	0.30	0.70
warped MSE	0.5	4.0	3.5
combined	0.5	4.5	4.0
NCC	0.99	0.30	0.69
MI	0.70	0.25	0.45
naive Corr	0.32	0	0.32
SSIM	0.99	0.90	0.09
PSNR	43 dB	28 dB	15 dB

**Table 4:** Per-pair runtime of each similarity metric (CPU, ms/pair).

Metric	Runtime (ms/Pair)
PSNR	1.44
MSE	1.59
Corr	2.60
NCC	3.97
MI	11.69
SSIM	24.70

## Data Availability

Radio-frequency data generated in this study can be made available under reasonable conditions.
